# Impact of Soft Liners on Bite Force in Complete Denture Wearers: A Prospective Cohort Study

**DOI:** 10.7759/cureus.77988

**Published:** 2025-01-25

**Authors:** Siddharth K Singla, Sandeep Kumar, Rajnish Aggarwal, Shabnam Choudhary, Sukhdeep Kaur, Sokhal Kartik, Pamei Jenthuilu

**Affiliations:** 1 Department of Prosthodontics, Surendera Dental College and Research Institute, Sri Ganganagar, IND

**Keywords:** bite force, complete dentures, conventional, edentulism, soft liners

## Abstract

Introduction: Edentulism is a significant global oral health concern, particularly among elderly individuals. Complete dentures are widely used to restore oral functionality, esthetics, and quality of life. The incorporation of soft liners into complete dentures has been proposed as a means of improving patient comfort and reducing the pressure on the oral mucosa. This study aimed to evaluate and compare the maximum bite force (MBF) in patients wearing complete dentures with and without soft liners for over six months.

Materials and methods: Forty-four completely edentulous patients participated in this prospective cohort study, divided into two groups: Group 1 (n=22) received complete dentures with long-term, heat-cured, plasticized acrylic liners (Permasoft, Dentsply International, York, PA, USA) and Group 2 (n=22) received conventional complete dentures without liners. MBF was recorded at two months (T0) and six months (T1) after denture delivery. Statistical analyses included paired t-tests for intragroup comparisons and independent t-tests for intergroup comparisons.

Results: Group 1 demonstrated a significant increase in MBF, from 46.07±12.28 N at T0 to 66.34±13.81 newtons (N) at T1 (P=0.021). Similarly, Group 2 exhibited an increase in MBF, from 38.32±12.61 N at T0 to 49.41±10.89 N at T1 (P=0.001). Intergroup comparisons revealed significantly higher MBF in Group 1 than in Group 2 at both time points, with a mean difference of 16.93 N (P=0.001) at T1.

Conclusion: The results indicated that the use of soft liners in complete dentures significantly increased MBF over six months compared with conventional dentures. These findings underscore the clinical value of soft liners in improving denture performance and patient outcomes.

## Introduction

Edentulism, or the complete loss of natural teeth, is a global oral health concern affecting millions of individuals, particularly the elderly population. Complete dentures are a widely adopted prosthetic solution for restoring oral functionality, esthetics, and quality of life in edentulous patients [1]. Notwithstanding their advantages, it is crucial to measure the occlusal force with full dentures to determine their chewing efficacy, which is contingent upon various factors, including the alignment of the dentures, the state of the supporting mucosal tissue, and the degree of patient acclimatization [2].

In addressing the challenges associated with traditional complete dentures, the incorporation of soft liners has emerged as a promising strategy to provide a cushioning intermediary between the inflexible denture base and oral mucosa, utilizing flexible materials such as silicone or acrylic composites. These liners are particularly effective in alleviating the pressure on mucosal surfaces, thereby enhancing patient comfort and improving retention. However, their effect on functional parameters, including occlusal force, remains a topic of ongoing investigation [3]. Babu et al. [4] concluded that the use of soft liners with complete dentures significantly reduced mandibular ridge resorption over a period of one year, compared to conventional denture wearers.

Bite force is a pivotal determinant in assessing the functional performance of dentures as it signifies a patient's capacity to effectively masticate food and sustain oral health. Previous studies have reported that 80% of conventional denture wearers experience pain while chewing food due to overloading of underlying soft tissues, complaining of masticatory insufficiency [5,6]. Although soft liners may provide improved comfort by reducing the pressure on underlying soft tissues, their long-term influence on bite force is still unclear. In a randomized controlled trial by Alqutaibi et al. [7], it was concluded that soft liners showed a positive effect on bite force and patient comfort over a period of three months. Therefore, by comparing bite force measurements in patients with complete dentures with and without soft liners, researchers can gain a deeper understanding of the clinical implications of soft liners and their role in optimizing prosthetic rehabilitation [8]. This study aimed to evaluate and compare the bite force in patients wearing complete dentures with and without the use of soft liners.

## Materials and methods

Study design and setting

This prospective cohort study was conducted in the Department of Prosthodontics at the Surendera Dental College and Research Institute, Sri Ganganagar, Rajasthan, India, from April 2023 to December 2023. Ethical committee approval was obtained from the Institutional Ethics Committee of Surendera Dental College and Research Institute (approval number: SDRI/IEC/22/35), and the study adhered to the principles of the Declaration of Helsinki. Written informed consent was obtained from all patients. All clinical and laboratory procedures were performed by a single operator to minimize variability.

Source of data

Convenience sampling was employed in this investigation, wherein patients attending the department for complete dentures were selected according to specific eligibility criteria. This was a prospective cohort study, as complete dentures were provided to patients based on their preferences (patients with previously ill-fitting dentures who chose complete dentures with soft liners for enhanced comfort and adaptability were categorized into Group 1, while others were assigned to Group 2), and the cohorts were monitored over a period of six months to assess bite force. Completely edentulous patients who desired a new set of complete dentures with a Class I ridge relationship with adequate interridge space and patients with good neuromuscular control were included in the study. Bruxers, patients with muscular dystrophy, patients without temporomandibular disorders (TMDs), and those who had difficulty understanding neurological or psychological problems were excluded from the study. The sample size was estimated to be 20 per group, using G*Power Version 3.6.9 (Heinrich-Heine-Universität Düsseldorf, Düsseldorf, Germany) at 90% power and 5% type 1 error for a clinically meaningful difference of 50 N in maximum bite force (MBF) at three months follow-up from a previous study [7]. Considering the 10% dropout rate, the present study was conducted in 22 patients per group. Therefore, 44 patients were included in the study.

Methodology

**Figure 1:**
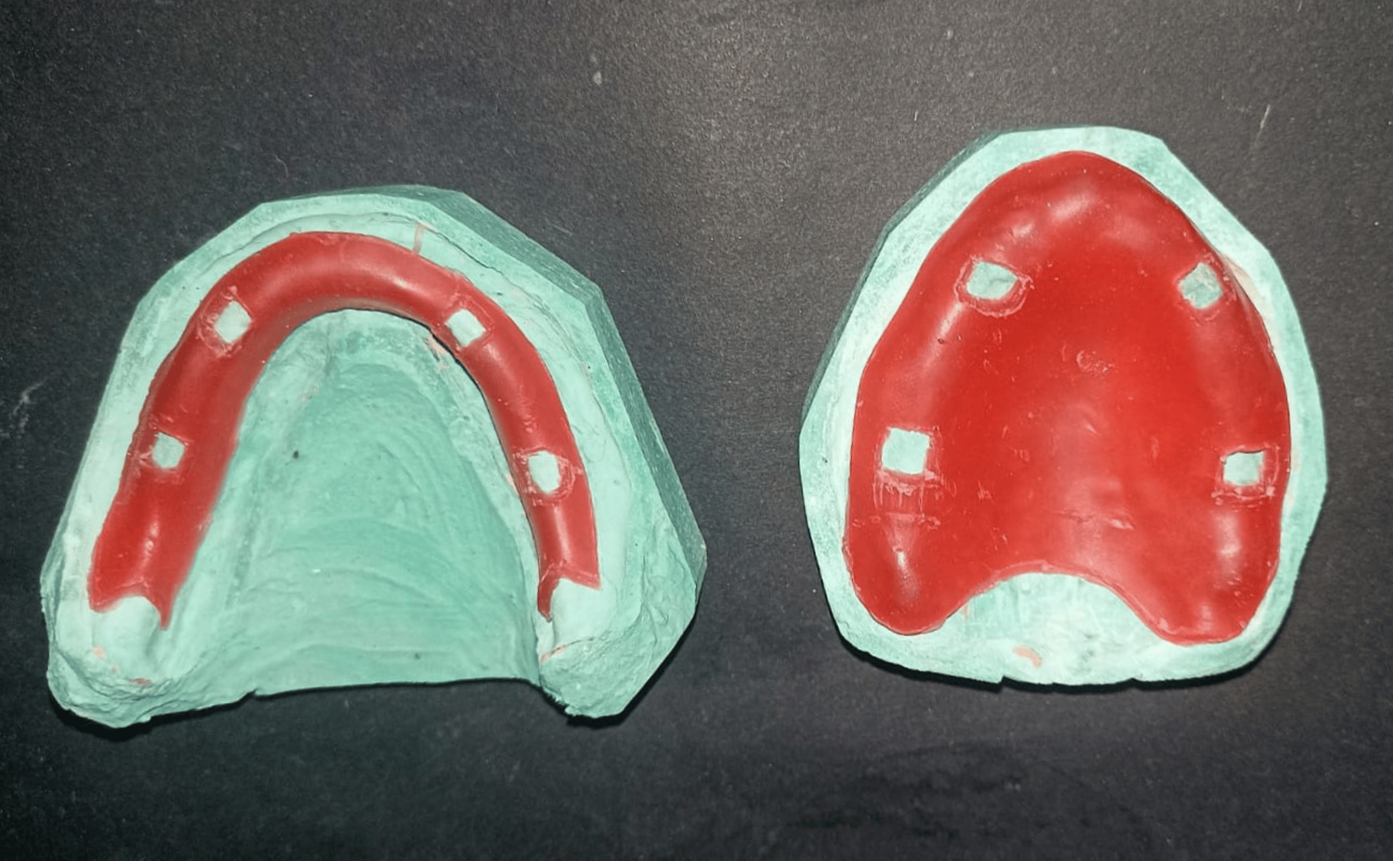
Wax spacer of 2 mm placed in fabricating complete denture with soft liner. Source: Derived from a study participant's case, with consent obtained for publication.

**Figure 2:**
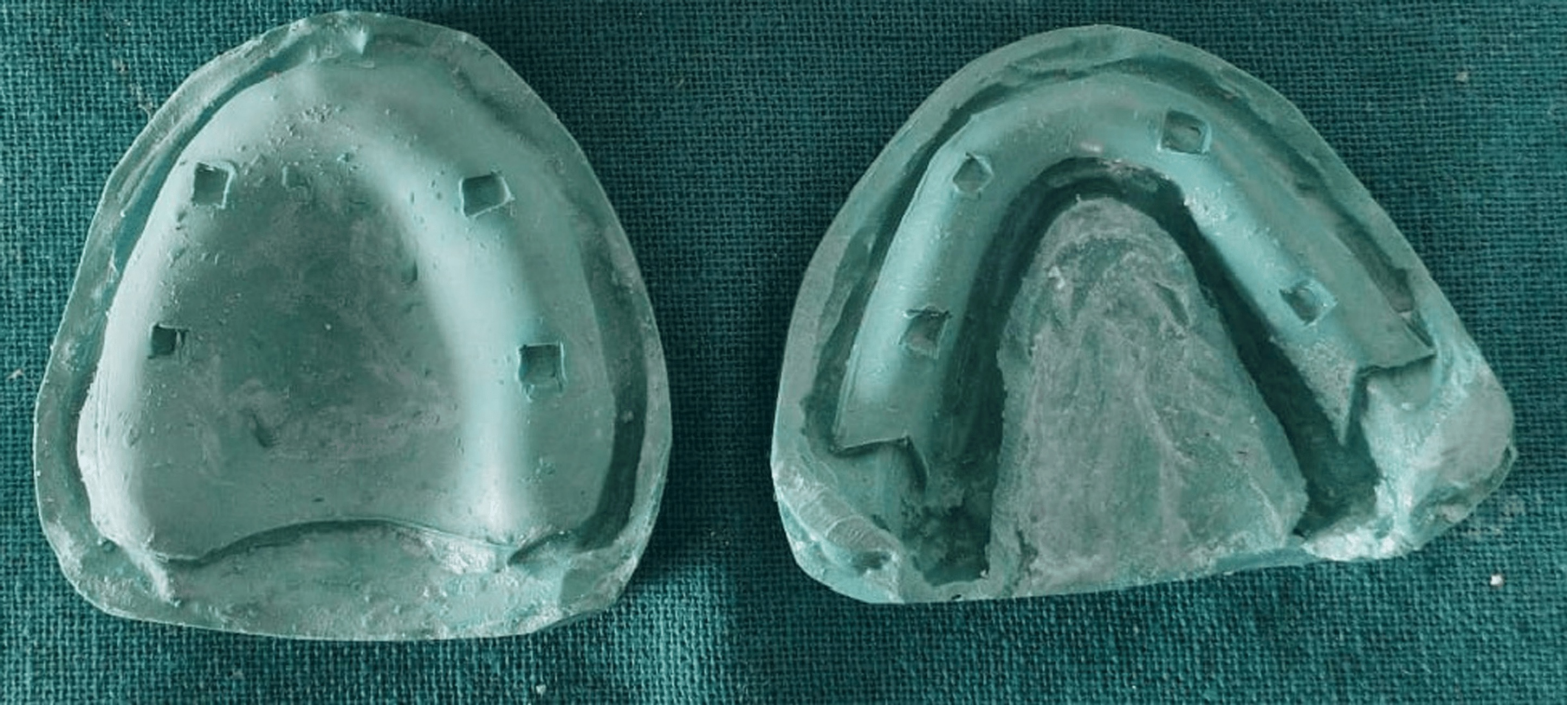
Duplicated casts for fabricating complete denture with soft liner. Source: Derived from a study participant's case, with consent obtained for publication.

The baseline characteristics were noted for all patients such as age, sex, body mass index (BMI), maxillary and mandibular ridge height, duration of edentulism, and duration of usage of the last denture. In Group 2, complete dentures were fabricated in a conventional manner using a conventional heat-activated denture base resin (DPI, Mumbai, India). In Group 1, complete dentures were fabricated by providing a 2-mm wax spacer on the master cast (Figure 1), which were then duplicated to fabricate complete dentures (Figure 2).

A long-term, heat-cured, plasticized acrylic liner (Permasoft, Dentsply International, York, PA, USA) was applied according to the manufacturer's instructions. Functional molding was performed on the chairside, followed by heat-cured polymerization, trimming, finishing, and polishing. After the delivery of the dentures, subsequent appointments for refinement were scheduled until the patients reported comfort and were free of any soft tissue irritation. In case of irritation, clinicians adjusted the dentures to relieve any discomfort. Furthermore, in instances where early occlusal contact was detected during mandibular closure, modifications to the occlusion were implemented using articulating papers.

**Figure 3:**
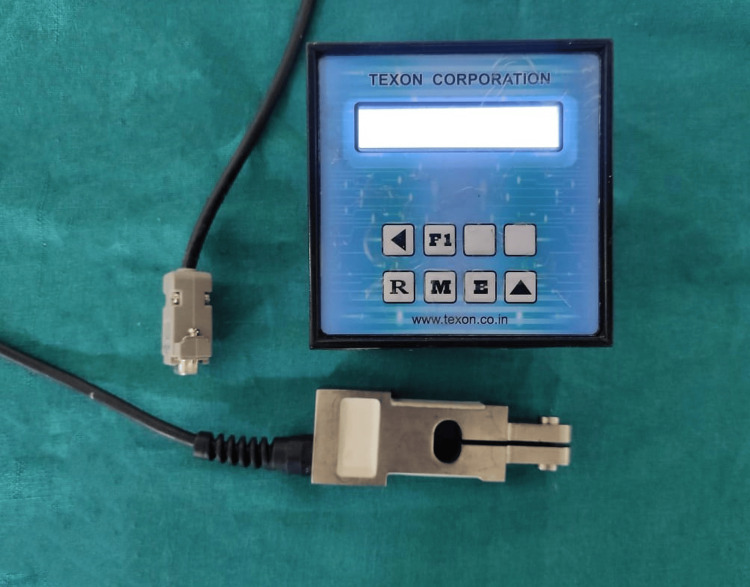
MBF was measured using a gnathodynamometer (Texon Co. Ltd., Uijeongbu-si, Republic of Korea). MBF: maximum bite force

Bite force evaluation was performed for all patients after two months of denture delivery, which was considered sufficient for masticatory adaptation in a previous study [9]. Bite force was measured using a gnathodynamometer (Texon Co. Ltd., Uijeongbu-si, Republic of Korea) after a period of two months (T0) and then after six months (T1), as shown in Figure 3. The device was itself properly calibrated and certified and was used according to the manufacturer's instructions.

**Figure 4:**
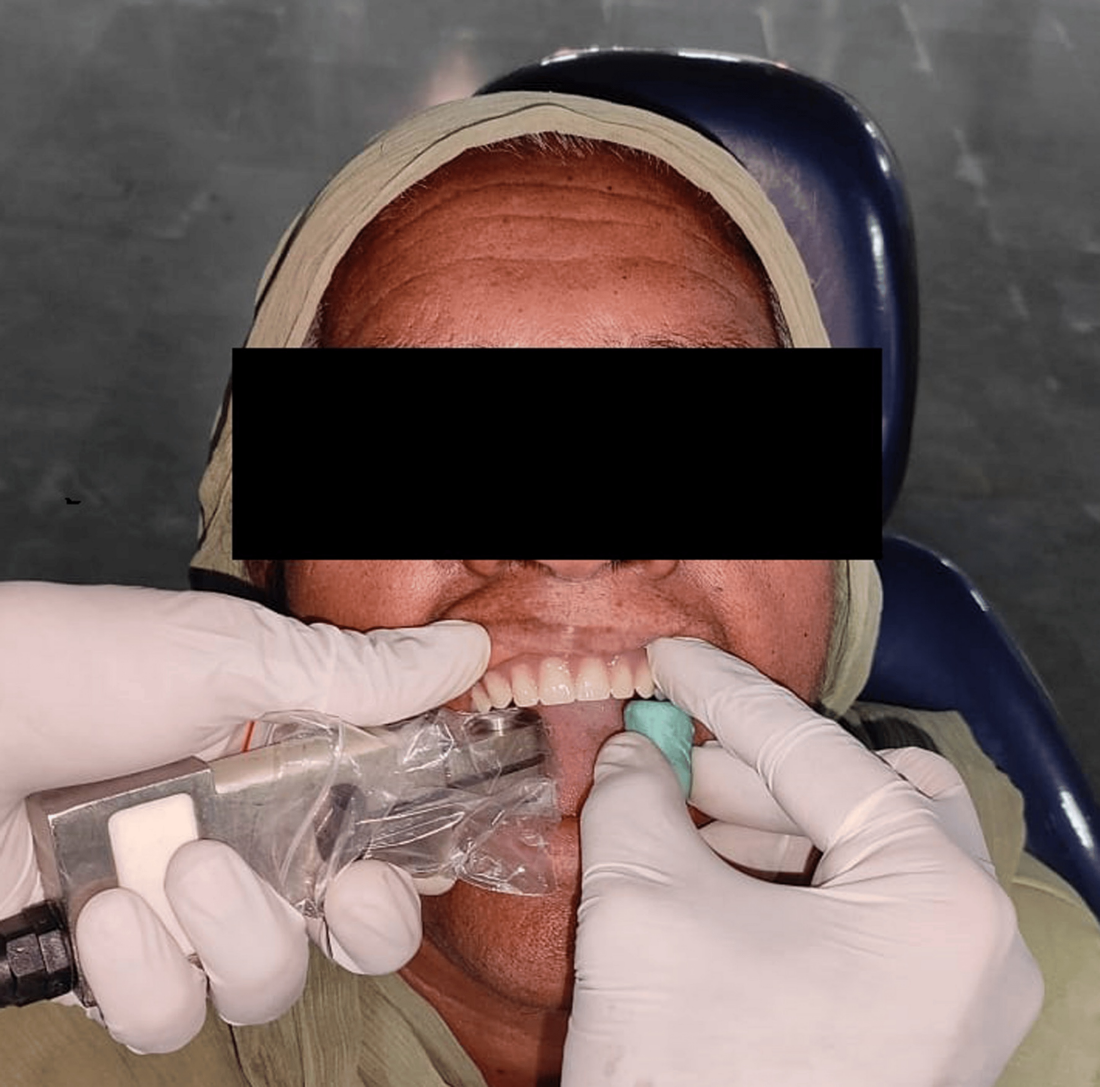
MBF was measured in the first molar area. MBF: maximum bite force Source: This figure represents image from a patient included in the study, used with consent.

An apparatus designed for the measurement of occlusal forces was employed to evaluate the bilateral MBF in the region of the first molars. The blocks utilized were constructed from additional silicone putty material (Chemzest Technoproducts Private Ltd., Chennai, India), matching the height of the measurement apparatus. These blocks were strategically positioned on the opposing side to mitigate any potential displacement of the dentures during the biting process (Figure 4).

While the apparatus was correctly positioned in relation to the first molar area, the patient was encouraged to apply MBF three times on each side, with a two-minute rest period between assessments. The mean MBF in newtons (N) for the three trials was calculated based on the participant's peak biting force, and the average value from both sides was established [7]. MBF was measured by an impartial evaluator who was unaware of the details pertaining to the interventions.

Statistical analysis

Statistical analysis was performed using the IBM SPSS Statistics for Windows, V. 23.0 (Released 2013; IBM Corp., Armonk, NY, USA). The Kolmogorov-Smirnov test was used to assess the normality of the data distribution. Data distribution with Q-Q plots confirmed normality. The intragroup comparison was performed using a paired t-test, and the intergroup comparison was performed using an independent Student's t-test. Sex differences at baseline were tested using the chi-squared (v2) test of independence. A statistical significance of P≤0.05 was determined to identify meaningful differences. The mean difference (MD) was calculated using the 95% confidence interval (CI).

## Results

As there was no loss to follow-up, the analysis was performed on 44 patients (22 in each group). The baseline characteristics of the study sample revealed non-significant differences between the groups (P>0.05), which showed that both groups were comparable at baseline, with a similar distribution of sex. The mean age of the patients in Group 1 was 67.82±4.21 years, whereas in Group 2, it was 66.12±5.19 years (Table 1).

**Table 1:** Baseline characteristics of the study sample. *: t-value by independent Student's t-test; **: t-value by the chi-squared test of independence; P>0.05: non-significant Group 1: complete denture with soft liners; Group 2: complete denture without soft liners Age, duration, height, and BMI are represented in the form of mean±SD, while sex is represented in the form of n (%). BMI: body mass index; SD: standard deviation

Variables	Group 1 (n=22)	Group 2 (n=22)	T-value or chi-squared value	P-value
Age in years	67.82±4.21	66.12±5.19	1.19*	0.24
Male (n (%))	8 (36%)	10 (45%)	5.81**	0.52
Females (n (%))	14 (64%)	12 (55%)	5.23**	0.87
Duration of edentulism in years	8.91±3.24	7.29±4.51	1.87*	0.18
Duration of the last denture usage in years	5.35±1.89	6.23±1.78	1.59*	0.12
Height of the upper alveolar ridge in mm	18.76±3.21	19.34±4.82	0.47*	0.64
Height of the lower alveolar ridge in mm	17.13±2.89	18.61±3.41	1.55*	0.13
BMI in kg/m^2^	20.89±3.24	21.24±4.04	0.32*	0.75

Group 1 showed an MBF of 46.07±12.28 N at two months, which increased to 66.34±13.81 N at six months follow-up. This difference was statistically significant (P=0.021). Similarly, in Group 2, the MBF was 38.32±12.61 N at two months, which increased to 49.41±10.89 N at six months follow-up. This difference was statistically significant (P=0.001) (Table 2).

**Table 2:** Intragroup comparison of MBF in N between the groups at different time intervals by paired t-test. *P≤0.05: significant Data is represented in the form of mean±SD. MBF: maximum bite force; N: newtons; SD: standard deviation; LL: lower limit; UL: upper limit; CI: confidence interval; MD: mean difference

Groups	MBF in N at two months (mean±SD)	MBF in N at six months (mean±SD)	MD at 95% CI (LL-UL)	T-value	P-value using paired t-test
Complete denture with soft liners (n=22)	46.07±12.28	66.34±13.81	20.27 (12.32-28.22)	2.432	0.021*
Complete denture without soft liners (n=22)	38.32±12.61	49.41±10.89	11.09 (3.92-18.26)	3.12	0.001*

Furthermore, the intergroup comparison showed that MBF values were higher in Group 1 than in Group 2 at both the observed time intervals, which showed that chewing efficiency was better with the use of soft liners and it increased with use (MD: 16.93; 95% CI: 9.36-24.49; P=0.001 at six months), as shown in Table 3.

**Table 3:** Intergroup comparison of MBF in N between the groups at different time intervals by independent Student’s t-test. *P≤0.05: significant Data is represented in the form of mean±SD. MBF: maximum bite force; N: newtons; SD: standard deviation; LL: lower limit; UL: upper limit; CI: confidence interval; MD: mean difference

Groups	Complete denture with soft liners (n=22)	Complete denture without soft liners (n=22)	MD at 95% CI (LL-UL)	T-value	P-value using independent t-test
MBF in N at two months (mean±SD)	46.07±12.28	38.32±12.61	7.75 (0.177-15.32)	2.06	0.045*
MBF in N at six months (mean±SD)	66.34±13.81	49.41±10.89	16.93 (9.36-24.49)	4.51	0.001*

## Discussion

The current investigation sought to assess the variations in MBF across two cohorts of patients over a duration of six months, with the use of soft liners in complete dentures compared to conventional dentures. The outcomes of this research underscore notable disparities in MBF between the two cohorts as well as intragroup changes over time. These findings offer significant implications for the functional advantages of soft liners in enhancing mastication efficiency and overall patient satisfaction. The comparable baseline characteristics lend credibility to the study design and strengthen the validity of our findings.

The masticatory force generated by natural dentition is estimated to be approximately 200 N; in contrast, the peak forces encountered during mastication by individuals utilizing complete dentures generally fall within the range of 60-80 N [10]. As articulated in a previous study, only 4% of the functional forces are effectively utilized, whereas the MBF values recorded during mastication do not exceed 22% of their theoretical potential [11]. Factors such as muscular strength and number of functional teeth are of paramount importance in the mastication process. The magnitude of bite force has been shown to have a significant correlation with patient satisfaction with complete dentures, the nature of the food ingested, and the extent of bone resorption that transpires beneath prosthetic appliances. Sex influences variations in the dynamics of biting; therefore, sex-based differences in MBF were not evaluated in the present study [12]. MBF was recorded in the first molar area, as nearly 80% of the force was found to be distributed in this area [13].

A statistically significant enhancement in MBF was noted in both cohorts throughout the six-month follow-up period. In Group 1, the MBF increased from 46.07±12.28 N at the two-month interval to 66.34±13.81 N at the six-month assessment. Pain on chewing due to the compressibility of complete dentures is the most common complaint of patients. This is more common in the lower arch (63%) [14]. Soft liners impart a cushioning effect, which is likely to enhance patient comfort and promote superior adaptation of dentures to the oral mucosa [15]. Over time, this augmented comfort and fit may have played a role in the increased masticatory efficiency and bite force observed.

Denture wearers exert only one-fifth to one-fourth of the occlusal force and masticatory efficiency compared to individuals with normal dentition [16]. Users of complete dentures tend to reach their occlusal force capacity earlier than those with natural teeth, which may be attributed to the pressure exerted by the denture base on the underlying mucosa, ultimately leading to the attainment of the pain threshold. Consequently, the elevated MBF observed with the use of soft liners can be ascribed to the elasticity of the liner, which alleviates stress on the alveolar ridge mucosa, along with a heightened pain threshold, which enhances the resilience of the alveolar ridge mucosa against stress [17].

Similarly, in Group 2, the MBF increased from 38.32±12.61 N at two months to 49.41±10.89 N at six months. While this improvement was statistically significant, the magnitude of change was less pronounced than that in Group 1. The observed improvements in MBF in Group 2 may be attributed to the natural adaptation of patients to their dentures over time, as well as improvements in neuromuscular coordination. Moreover, with time, the inhibitory impact of psychological factors is minimized, which ensures imminent acceptance of complete dentures [18]. Similar results were reported in previous studies [8,9,14,17].

The authors used long-term, heat-cured, plasticized acrylic liners, which have been shown to display high MBF over conventional dentures over a follow-up period of three months [7]. Similar results were reported in a study by Bahl et al. [19], who found significantly less mandibular ridge resorption in patients where complete dentures were given along with long-term resilient liners, compared to conventional dentures, over a period of one year after denture delivery.

Clinical implications

The results of this study have important clinical implications for prosthodontic practice. The significant improvements in MBF observed in the soft-liner group suggest that soft liners may be particularly beneficial for patients experiencing discomfort or difficulty adapting to traditional dentures. The enhanced chewing efficiency associated with soft liners can improve dietary intake and nutritional status, which is especially critical for the elderly or medically compromised individuals. Moreover, the use of soft liners may reduce the need for frequent denture adjustments and relining, thereby improving long-term patient satisfaction. As the benefits of soft liners are sustained with continued use, their long-term utility in improving denture performance and patient outcomes is advisable. However, clinicians should consider the potential drawbacks of soft liners, such as their increased susceptibility to fungal infections, degradation over time, and the need for regular maintenance. Patient education on proper care and hygiene of soft-lined dentures is essential to mitigate these risks and maximize the benefits of this intervention.

Strengths and limitations

The strengths of this study include its robust design, comparable baseline characteristics, and well-defined follow-up period. The use of objective measures, such as MBF, enhances the reliability of the findings. Additionally, the absence of loss to follow-up ensured the completeness of the data and minimized the risk of bias.

However, this study also has limitations that should be considered when interpreting the results. The relatively small sample size (44 patients) may limit the generalizability of our findings to broader populations. Future studies with larger sample sizes and diverse patient groups are needed to confirm these results and to explore the effects of soft liners in different clinical contexts. Additionally, the study did not assess patient-reported outcomes such as comfort, satisfaction, and quality of life, which are critical aspects of prosthodontic care. Incorporating these measures into future research would provide a more comprehensive understanding of the benefits of soft liners. Another limitation is the relatively short follow-up period of six months. While this study demonstrated significant improvements in MBF during this period, longer-term studies are needed to evaluate the durability of these benefits and the potential effects of wear and tear on soft liners. Moreover, sex-based differences in MBF were not evaluated in this study.

## Conclusions

The findings of this study highlight the significant advantages of soft liners in improving MBF in denture wearers. The observed improvements in MBF with soft liners at the two- and six-month follow-up periods were both statistically and clinically significant, underscoring their potential to improve dietary intake, nutritional status, and overall quality of life. Further research is needed to evaluate the long-term outcomes, cost-effectiveness, and patient-reported benefits of this intervention as well as to explore strategies for addressing potential drawbacks such as microbial colonization and material degradation.

## References

[1] Emami E, de Souza RF, Kabawat M, Feine JS (2013). The impact of edentulism on oral and general health. Int J Dent.

[2] Fontijn-Tekamp FA, Slagter AP, Van Der Bilt A, Van 'T Hof MA, Witter DJ, Kalk W, Jansen JA (2000). Biting and chewing in overdentures, full dentures, and natural dentitions. J Dent Res.

[3] Hashem MI (2015). Advances in soft denture liners: an update. J Contemp Dent Pract.

[4] Babu BD, Jain V, Pruthi G, Mangtani N, Pillai RS (2017). Effect of denture soft liner on mandibular ridge resorption in complete denture wearers after 6 and 12 months of denture insertion: a prospective randomized clinical study. J Indian Prosthodont Soc.

[5] Żmudzki J, Chladek G, Kasperski J (2015). Biomechanical factors related to occlusal load transfer in removable complete dentures. Biomech Model Mechanobiol.

[6] Limpuangthip N, Somkotra T, Arksornnukit M (2021). Subjective and objective measures for evaluating masticatory ability and associating factors of complete denture wearers: a clinical study. J Prosthet Dent.

[7] Alqutaibi AY, Alnazzawi AA, Farghal AE, Bakr RM, Mahmoud II (2023). Impact of acrylic and silicone-based soft-liner materials on biting force and quality of life of the complete denture wearers: a randomized clinical trial. J Clin Med.

[8] Kimoto S, Yamamoto S, Shinomiya M, Kawai Y (2010). Randomized controlled trial to investigate how acrylic-based resilient liner affects on masticatory ability of complete denture wearers. J Oral Rehabil.

[9] Kimoto S, So K, Yamamoto S, Ohno Y, Shinomiya M, Ogura K, Kobayashi K (2006). Randomized controlled clinical trial for verifying the effect of silicone-based resilient denture liner on the masticatory function of complete denture wearers. Int J Prosthodont.

[10] Hickey JC, Kreider JA, Boucher CO, Storz O (1989). A method of studying the influence of occlusal schemes on muscular activity. J Pros Dent.

[11] Iwaki M, Kanazawa M, Soeda Y, Hada T, Komagamine Y, Minakuchi S (2024). Effect of digital complete dentures manufactured using the custom disk method on masticatory function. Heliyon.

[12] Wichelhaus A, Hüffmeier S, Sander FG (2003). Dynamic functional force measurements on an anterior bite plane during the night. J Orofac Orthop.

[13] Tortopidis D, Lyons MF, Baxendale RH, Gilmour WH (1998). The variability of bite force measurement between sessions, in different positions within the dental arch. J Oral Rehabil.

[14] Kimoto S, Kimoto K, Gunji A (2007). Clinical effects of acrylic resilient denture liners applied to mandibular complete dentures on the alveolar ridge. J Oral Rehabil.

[15] Saeed F, Muhammad N, Khan AS, Sharif F, Rahim A, Ahmad P, Irfan M (2020). Prosthodontics dental materials: from conventional to unconventional. Mater Sci Eng C Mater Biol Appl.

[16] Michael CG, Javid NS, Colaizzi FA, Gibbs CH (1990). Biting strength and chewing forces in complete denture wearers. J Prosthet Dent.

[17] Shala K, Tmava-Dragusha A, Dula L, Pustina-Krasniqi T, Bicaj T, Ahmedi E, Lila Z (2018). Evaluation of maximum bite force in patients with complete dentures. Open Access Maced J Med Sci.

[18] Turker SB, Sener ID, Özkan YK (2009). Satisfaction of the complete denture wearers related to various factors. Arch Gerontol Geriatr.

[19] Bahl J, Gill HS, Kapoor N, Nagpal A, Verma R (2023). The effect of two heat cure soft tissue liners on mandibular ridge resorption at various time intervals after complete denture Insertion. Int J Drug Res Dental Sci.

